# Succinic acid production with *Actinobacillus succinogenes*: rate and yield analysis of chemostat and biofilm cultures

**DOI:** 10.1186/s12934-014-0111-6

**Published:** 2014-08-19

**Authors:** Hendrik Gideon Brink, Willie Nicol

**Affiliations:** Department of Chemical Engineering, University of Pretoria, Lynnwood Road, Hatfield, Pretoria, 0002 South Africa

**Keywords:** *Actinobacillus succinogenes*, Succinic acid, Biofilm reactor, Chemostat, Continuous culture, Maintenance kinetics, Metabolic flux distribution

## Abstract

**Background:**

Succinic acid is well established as bio-based platform chemical with production quantities expecting to increase exponentially within the next decade. *Actinobacillus succinogenes* is by far the most studied wild organism for producing succinic acid and is known for high yield and titre during production on various sugars in batch culture. At low shear conditions continuous fermentation with *A. succinogenes* results in biofilm formation. In this study, a novel shear controlled fermenter was developed that enabled: 1) chemostat operation where self-immobilisation was opposed by high shear rates and, 2) *in-situ* removal of biofilm by increasing shear rates and subsequent analysis thereof.

**Results:**

The volumetric productivity of the biofilm fermentations were an order of magnitude more than the chemostat runs. In addition the biofilm runs obtained substantially higher yields. Succinic acid to acetic acid ratios for chemostat runs were 1.28±0.2 g.g^-1^, while the ratios for biofilm runs started at 2.4 g.g^-1^ and increased up to 3.3 g.g^-1^ as glucose consumption increased. This corresponded to an overall yield on glucose of 0.48±0.05 g.g^-1^ for chemostat runs, while the yields varied between 0.63 g.g^-1^ and 0.74 g.g^-1^ for biofilm runs. Specific growth rates (μ) were shown to be severely inhibited by the formation of organic acids, with μ only 12% of μ_max_ at a succinic acid titre of 7 g.L^-1^. Maintenance production of succinic acid was shown to be dominant for the biofilm runs with cell based production rates (extracellular polymeric substance removed) decreasing as SA titre increases.

**Conclusions:**

The novel fermenter allowed for an in-depth bioreaction analysis of *A. succinogenes.* Biofilm cells achieve higher SA yields than suspended cells and allow for operation at higher succinic acid titre. Both growth and maintenance rates were shown to drastically decrease with succinic acid titre. The *A. succinogenes* biofilm process has vast potential, where self-induced high cell densities result in higher succinic acid productivity and yield.

## Background

Succinic acid (SA) is well established as a bio-based platform chemical and intermediate [1]. The terminal carboxylic acid groups open up numerous possibilities for further processing. Major developments include polymerisation of SA with its hydrogenated diol product (1,4-butanediol) to produce the biodegradable plastic polybutylene succinate [2]. Another application is the use of 1,4-butanediol to produce tetrahydrofuran, an intermediate for the production of elastic fibres and thermoplastics [3]. SA also poses the option of replacing the traditional petrochemical market of maleic anhydride, due to its similarity in chemical structure [4]. All these developments have the prospect of high-volume production and accordingly the efficient production of SA from renewable resources has become a topical challenge.

All bio-based SA is produced via fermentation. Bacterial strains, both wild and genetically modified, represent the overwhelming majority of open literature publications [5]. *Actinobacillus succinogenes* is by far the most prominent wild strain in these studies, while *Escherichia coli* is the preferred organism for manipulations of the central carbon metabolism. SA fermentation allows for unusually high product yields on carbon substrate due to the carbon dioxide fixation step [6]. In theory, it is possible to obtain a yield of 1.12 gram SA per gram glucose consumed (Y_Glc,SA_) if biomass formation is ignored [7]. Preliminary batch runs on modified *E. coli* strains have reported Y_Glc,SA_ in excess of 1 g.g^−1^ [8,9], while a maximum Y_Glc,SA_ of 0.94 g.g^−1^ has been reported for *A. succinogenes* [10].

*A. succinogenes* remains an attractive production strain. It has been shown to metabolise most naturally occurring sugars [11] and to produce SA at a titre close to the saturation point (>95 g.L^−1^) [12], while volumetric productivities in excess of 10 g.L^−1^.h^−1^ have been reported [13]. There are more than one hundred open literature publications on the strain, with the majority employing batch fermenters and various substrates. From a processing perspective, high cell density fermentation can be described as a requirement in order to enhance volumetric productivity and subsequently reduce capital expenses. This requires a cell retainment strategy where cells are separated from the fermentation broth and concentrated in the fermenter. *A. succinogenes* is well known to self-adhere to support surfaces and form biofilms under prolonged operation. All continuous studies on *A. succinogenes* resulted in unavoidable biofilm formation [7,13–15] except the study by Kim *et al.* [16], where a membrane separation recycle system was implemented. The limited fermentation times in the Kim study was most probably caused by biofilms blocking the filter. Accordingly, self-immobilisation is the only cell retainment option for achieving high cell densities. The biofilm mode of operation is not limited to continuous fermenters and can also be employed in repeat batch fermentations where the attached biomass is retained after a batch cycle is completed [12,17]. In addition, the repeat batch fermentation can be supplemented with substrate (fed-batch) during the fermentation cycle [11].

**Figure 1:**
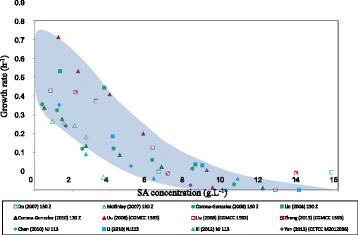
**Growth rates vs. C**_**SA**_ **of prominent batch studies on** ***A. succinogenes*** **employing Glc as substrate [** 12 **,** 18 **-** 27 **].** The μ values were estimated from the reported biomass/SA vs. time profiles. Severe product inhibition is evident with growth ceasing between 8 g.L^−1^ and 14 g.L^−1^ of SA. The blue data cloud covers the majority of the experimental measurements.

Kinetic analyses of *A. succinogenes* are limited to batch fermentations in the absence of cell immobilisation [18,19] since biofilms only form after prolonged operation (typically under continuous conditions). Numerous authors have reported batch profiles using various substrates (see Figure 1 for notable studies on glucose (Glc) [12,18–27]). All these studies are characterised by a point in time where cell growth terminates while metabolite production continues beyond the termination point. In Figure 1, some of the prominent studies using Glc are represented where the specific growth rates (μ), estimated from biomass curves, are plotted against SA titre. All these experiments were started without addition of SA to the medium and with different medium formulations. SA is chosen as the indicator for growth inhibition although all metabolically produced acids (SA, acetic acid (AA) and formic acid (FA)) are known to contribute to inhibition [22]. The catabolite ratios vary to some extent and SA can only be used as a relative indicator. Most of the data in Figure 1 fall within the blue ‘data cloud’ and drastic growth inhibition is reflected by lower growth rates between 8 and 14 g.L^−1^ of SA. Surprisingly, the SA titre in Figure 1 does not correspond to the terminal SA titre when succinate is externally added prior to the fermentation [22,28]. In both these studies, growth was observed above SA titres of 30 g.L^−1^ indicating a difference between the influence of external and produced succinate on *A. succinogenes*. Another striking similarity between the various batch runs is that the production rates of the by-products (AA and FA) decrease after the growth termination point while SA production appears to be unaffected. This clearly indicates that non-growth or maintenance production of SA plays an important role and that yield differences exist between the growth and maintenance modes. In some studies, the consumption of FA is reported in the non-growth phase of the batch fermentation [24,27,29]. With regard to substrate inhibition, differences between biofilm and suspended cultures are reported [12] where established biofilms appear to be unaffected by the initial Glc concentration while the lag time of the suspended cultures (no lag for biofilm cultures) is clearly influenced by the initial Glc concentrations, with higher lag times reported for higher Glc concentrations [18].

Given the economic requirement for high cell density fermentation, more insight is required on the rate and yield characteristics of *A. succinogenes* biofilms. Biofilm studies on *A. succinogenes* [7,13–15] only reported overall performance and product distributions and did not attempt a cell based description of the conversion process. The main reason for this is that the quantification of attached biomass is much harder and more time consuming than quantifying suspended biomass. For the continuous fermenter without biofilm (chemostat), cell quantification can be performed by mere analysis of the fermenter effluent, while the biofilm fermenter requires the removal of all attached cells, where termination of the run is typically required. In the study by Maharaj, Bradfield & Nicol [13], a limited number of biofilm removal experiments were performed and an indication was given that total biomass activity decreased drastically with an increase in the metabolite concentration in the broth. Total biomass analysis is complicated by the presence of extracellular polymeric substance (EPS) where the unreactive fraction of the total biomass (i.e. EPS) is unknown. Further complexities can also arise from product gradients within the biofilm where cells deeper in the biofilm matrix might contribute less to production of metabolites. It is clear that more fundamental insight is required in order to develop an optimal biofilm process and the present study therefore focuses on this objective.

In this work, a novel, purpose-specific fermenter was employed to investigate the steady-state rate and yield characteristics of *A. succinogenes* biofilms. Steady-state is ideal for kinetic and yield analyses as stable operation allows for mass balance checks. The fermenter comprised a tubular, silicone recycle reactor where biofilm attached to the internal surface of the tube. The flowrate through the tube could be adjusted and ensured a uniform distribution of shear on the external biofilm surface. High shear rates allowed for *in-situ* removal of biofilm and the subsequent analysis of the total biomass without termination of the fermentation. The fermenter also allowed for chemostat operation (only suspended cells) by maintaining high shear rates therefore preventing cell attachment. This allowed for a proper growth analysis of the continuous culture and assisted in interpreting the continuous biofilm results. Removed biofilm was treated by alkaline hydrolysis to separate EPS from cells, which meant that it was possible to use only the metabolically active biomass as the basis for the rate analysis.

## Results and discussion

### Chemostat analysis

**Figure 2:**
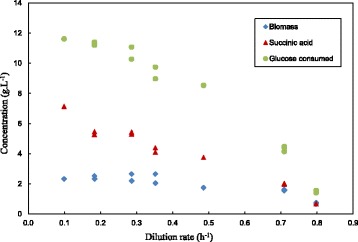
**Chemostat steady-state measurements of ΔGlc, SA and biomass plotted against D.** High shear conditions prevented biomass attachment. Extrapolation of data indicates that μ_max_ lies between 0.8 h^−1^ and 0.85 h^−1^.

All steady-state chemostat data are given in Table 1. Dilution rates (D) varied between 0.1 h^−1^ and 0.8 h^−1^. The Glc consumed (ΔGlc), SA titre and biomass (x) in the outlet are plotted as a function of D in Figure 2. Repeat runs were performed at five of the seven conditions and is indicated in Table 1 and Figure 2. For these runs, the average deviation in the SA titre was 3.0% with a maximum observed deviation of 7.2%. Mass balance checks at steady-state conditions (assuming a biomass composition of CH_1.8_O_0.5_ N_0.2_) indicated that the residual Glc and metabolites in the fermenter outlet accounted for more mass than that determined from the ΔGlc. The average overestimation of 16% (with standard deviation of 10%) indicates that a significant fraction of constituents from yeast extract (YE) and/or corn steep liquor (CSL) were incorporated into the formed biomass. The decline in the ΔGlc with an increase in D in Figure 2 suggests that washout will occur between a D of 0.8 h^−1^ and 0.85 h^−1^, suggesting a maximum specific growth rate (μ_max_) within this range.

**Table 1:** **Chemostat steady-state results obtained during high shear experiments**

***Run no.***	***D (h*** ^***−1***^ ***)***	***Glc out (g.L*** ^***−1***^ ***)***	***ΔGlc (g.L*** ^***−1***^ ***)*** ^*1^	***SA (g.L*** ^***−1***^ ***)***	***AA (g.L*** ^***−1***^ ***)***	***FA (g.L*** ^***−1***^ ***)***	***Suspended x (g.L*** ^***−1***^ ***)***	***SA/AA (g.g*** ^***−1***^ ***)***	***FA/AA (g.g*** ^***−1***^ ***)***	***Y***_***Glc,SA***_ ***(g.g*** ^***−1***^ ***)***	***q***_***SA***_ ***(g.L*** ^***−1***^ ***h*** ^***−1***^ ***)***	***r***_***SA***_ ***(g.g*** ^***−1***^ ***h*** ^***−1***^ ***)***
*1*	*0.10*	*27.7*	*11.61*	*7.14*	*5.24*	*0.04*	*2.33*	*1.36*	*0.01*	*0.61*	*0.71*	*0.30*
*2*	*0.18*	*31.1*	*11.41*	*5.28*	*4.44*	*0.97*	*2.53*	*1.19*	*0.22*	*0.46*	*0.97*	*0.38*
*3*	*0.18*	*31.2*	*11.20*	*5.48*	*3.65*	*1.22*	*2.33*	*1.50*	*0.33*	*0.49*	*1.00*	*0.43*
*4*	*0.29*	*31.5*	*10.27*	*5.31*	*3.42*	*1.93*	*2.19*	*1.55*	*0.56*	*0.52*	*1.52*	*0.69*
*5*	*0.29*	*30.7*	*11.08*	*5.44*	*3.77*	*1.82*	*2.65*	*1.44*	*0.48*	*0.49*	*1.56*	*0.59*
*6*	*0.35*	*32.7*	*9.74*	*4.40*	*3.29*	*1.96*	*2.65*	*1.34*	*0.60*	*0.45*	*1.55*	*0.58*
*7*	*0.35*	*33.5*	*8.97*	*4.10*	*2.70*	*1.77*	*2.04*	*1.52*	*0.66*	*0.46*	*1.44*	*0.71*
*8*	*0.49*	*34.4*	*8.53*	*3.76*	*3.10*	*1.54*	*1.74*	*1.22*	*0.50*	*0.44*	*1.83*	*1.05*
*9*	*0.71*	*34.9*	*4.15*	*1.97*	*1.62*	*1.19*	*1.55*	*1.21*	*0.73*	*0.48*	*1.40*	*0.90*
*10*	*0.71*	*34.6*	*4.47*	*1.97*	*1.71*	*1.19*	*1.62*	*1.15*	*0.69*	*0.44*	*1.40*	*0.86*
*11*	*0.71*	*34.6*	*4.41*	*2.03*	*1.64*	*1.20*	*1.57*	*1.23*	*0.73*	*0.46*	*1.44*	*0.92*
*12*	*0.80*	*38.3*	*1.41*	*0.68*	*0.73*	*0.32*	*0.67*	*0.93*	*0.44*	*0.48*	*0.54*	*0.81*
*13*	*0.80*	*38.2*	*1.57*	*0.69*	*0.74*	*0.35*	*0.76*	*0.94*	*0.47*	*0.44*	*0.55*	*0.73*

^*1^Effect of dilution due to NaOH dosing incorporated into the calculation.

**Figure 3:**
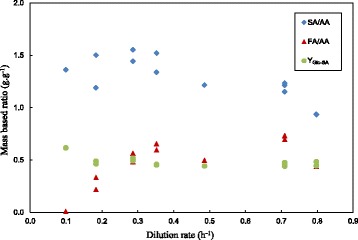
**Chemostat mass-based yield ratios (SA/AA, FA/AA and Y**_**Glc,SA**_ **) plotted against D.** The average Y_Glc,SA_ is relatively stable at 0.48 ± 0.047 g.g^−1^. FA/AA is close to the equimolar value (0.77 g.g^−1^) at a D of 0.8 h^−1^, decreasing to zero as D is decreased. SA/AA is in the vicinity of 1.4 g.g^−1^ for D values below 0.5 h^−1^, while a slight decrease is observed at higher D values.

The chemostat product distribution can be seen in Figure 3 where Y_Glc,SA_ as well as the by-product ratios are plotted. Overall Y_Glc,SA_ values are fairly constant around the average value of 0.48 ± 0.047 g SA.(g ΔGlc)^−1^. The SA to AA ratio (SA/AA) was in the vicinity of 1.4 g.g^−1^ for D values below 0.5 h^−1^ while a slight decrease in the value is observed at higher D values. The formation of FA decreased at lower values of D where a negligible amount of FA was observed at D = 0.1 h^−1^. The maximum value of the FA to AA ratio (FA/AA) of 0.73 g.g^−1^ obtained at a high D is close to the equimolar value of 0.77 g.g^−1^. This implies that the pyruvate formate lyase route for the oxidation of pyruvate is dominant at high D. The decrease in the FA/AA ratio with a reduction in D is linked to either pyruvate dehydrogenase or formate dehydrogenase contributing to the formation of less FA. The FA/AA decrease corresponds to the observations of Bradfield & Nicol [15] where low D in a biofilm reactor resulted in a similar trend. The decrease in FA is most likely attributed to formate dehydrogenase converting FA to CO_2_ and NADH since Zheng *et al.* [24], Xi *et al.* [27] and Du *et al.* [29] observed a FA decrease against time after initial formation in a batch fermenter.

The SA/AA observed in Figure 3 is lower than the expected values when redox closure of the catabolic pathways is considered, while ignoring the anabolic pathways responsible for biomass synthesis. The expected values reported by Bradfield & Nicol [15] suggest that the SA/AA should vary between 1.97 g.g^−1^ (for FA/AA = 0.77 g.g^−1^) and 3.93 g.g-1 (for FA/AA = 0 g.g^−1^). The slight increase in the SA/AA at lower D is likely linked to the decrease in the FA/AA ratio although the values are far below the expected ratios. This suggests that significant amounts of NADH are consumed in the anabolism since direct oxidation of NADH under anaerobic conditions is unlikely. Although biomass synthesis utilising a complex medium is considered to be redox neutral [30], similar observations have been made for growth of *Lactobacillus rhamnosus* in a YE rich medium by the authors of this study [31].

**Figure 4:**
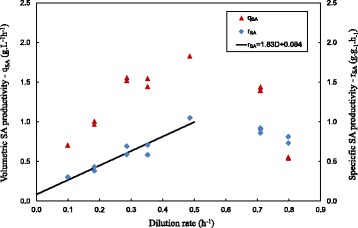
**Chemostat SA productivities (q**_**SA**_ **and r**_**SA**_ **) plotted against D.** The r_SA_ vs. D curve is expected to follow a straight line fit with the slope giving Y_x,SA_^true^ and the intercept giving m_SA_. The straight line trend is observed for all values except the highest values of D. This is most likely connected to inaccurate measurements at low biomass concentrations, where small amounts of insoluble material inflate the measurements. Accordingly, only the r_SA_ measurements at the D values between 0.1 h^−1^ and 0.5 h^−1^ were considered for the linear regression. Regression model given by equation (1).

In Figure 4, the SA volumetric productivity (q_SA_) and SA specific productivity (r_SA_), i.e. based on cell mass, are given. The q_SA_ obeys the expected behaviour by reaching a maximum of 1.8 g.L^−1^ h^−1^ at an intermediate D of 0.5 h^−1^. The specific productivity is expected to fit a straight line where the slope gives the growth associated yield coefficient of SA on biomass (Y_x,SA_^true^) [30]. The trend is observed except for the highest D values of 0.7 h^−1^ and 0.8 h^−1^ where a lower r_SA_ is observed. This is most likely attributed to inaccurate biomass measurements at low biomass concentrations, where small amounts of insoluble material present in the medium inflate the measurements. Accordingly, only readings from D = 0.1 h^−1^ to D = 0.5 h^−1^ were considered for the linear regression. The straight line fit is given as Equation (1) and corresponds well with batch data by Corona-González et al. on the same strain of *A. succinogenes* [19,22].

1 $$ {r}_{SA}=1.83D+0.084 $$

Since maintenance or non-growth production of SA is considered to be prominent under conditions where cell growth has ceased (see Background), it is important to establish the maintenance contribution concurrent with growth that is prevalent in the chemostat. The estimate of maintenance production of SA (m_SA_) can be obtained from the y-axis intersection of the linear fit on Figure 4 [30]. The estimated value of m_SA_ = 0.084 g.g^−1^ is small when compared to the total SA production rate represented by the blue diamonds. Even at the lowest D of 0.1 h^−1^ where growth inhibition is most severe the maintenance only accounts for 27% of the total SA production.

**Figure 5:**
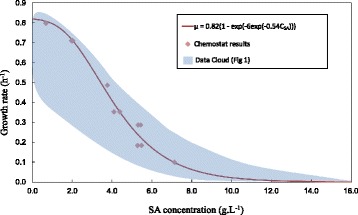
**Chemostat growth rates (μ) plotted against SA concentration.** Good agreement with the data cloud in Figure 1 is obtained. Inhibition model given by equation (2) with μ_max_ at 0.82 h^−1^.

In order to quantify the growth inhibition, μ (or D for a chemostat) is plotted against the SA titre in Figure 5 and compared to the data cloud given in Figure 1. It should be noted that SA is not the only inhibitor and that the other acids (AA and FA) also contribute to growth inhibition [22]. Due to the observation that the by-product relationships exhibit a consistent trend with D, the choice of the inhibition variable is irrelevant. The chemostat data compares remarkably well with batch data from literature, with the severity of inhibition clearly evident. Equation (2) represents an empirical fit (r^2^ = 0.98) of the chemostat data using a Gompertz asymmetrical sigmoid function [32] which incorporates the μ_max_ of 0.82 h^−1^ and the gradual tailing of the data cloud beyond the highest measured SA titre (C_SA_) of this study.

2 $$ \mu =0.82\left(1- \exp \left(-6.0 \exp \left(-0.54{C}_{SA}\right)\right)\right) $$

It is evident from Figure 5 that growth inhibition is severe since the growth rate is reduced eightfold by merely increasing the SA titre from 0 g.L^−1^ to 7 g.L^−1^. The asymptotic behaviour of the inhibition function beyond a SA titre of 14 g.L^−1^ is uncertain but it is evident that growth beyond this point is extremely slow. This supports the notion presented by Maharaj *et al.* [13] that high titre production of SA (SA > 15 g.L^−1^) is predominantly maintenance driven.

### Biofilm analysis

For the biofilm runs, eleven separate steady-states were achieved and for all of these runs the total biomass content was quantified. All the measurements from the eleven steady-states are reported in Table 2. For most of the steady-states the suspended biomass was not detected and a clear effluent was observed. Significant biomass concentrations were observed in run 3 and 10. These occurrences were unrelated to D and rather associated with natural biofilm shedding phases. Mass balance checks on steady-state conditions fell to within 90-102% (accounted mass from outlet divided by stoichiometric equivalent inlet mass). These results suggest that the incorporation of amino acids and peptides (from YE and CSL) to form new biomass is minimal at steady-state conditions, hinting that growth plays an insignificant role for the biofilm runs. Note that the biofilm build-up is gradual and that the majority of the biofilm is a former metabolic product that developed before steady-state was achieved. Accordingly the only biomass considered in the mass balance check is the suspended biomass which is assumed to form continuously at steady-rate.

**Table 2:** **Biofilm steady-state results obtained during low shear experiments**

***Run no.***	***D (h*** ^***−1***^ ***)***	***Glc out (g.L*** ^***−1***^ ***)***	***ΔGlc (g.L*** ^***−1***^ ***)*** ^*1^	***SA (g.L*** ^***−1***^ ***)***	***AA (g.L*** ^***−1***^ ***)***	***FA (g.L*** ^***−1***^ ***)***	***Suspended x (g.L*** ^***−1***^ ***)***	***Total x (g.L*** ^***−1***^ ***)*** ^*2^	***Fraction cells in total x***	***SA/AA (g.g*** ^***−1***^ ***)***	***FA/AA (g.g*** ^***−1***^ ***)***	***Y***_***Glc,SA***_ ***(g.g*** ^***−1***^ ***)***	***q***_***SA***_ ***(g.L*** ^***−1***^ ***h*** ^***−1***^ ***)***	***r***_***SA***_ ***(g.g*** ^***−1***^ ***h*** ^***−1***^ ***)*** ^*3^	***Modified r***_***SA***_ ***(g.g*** ^***−1***^ ***h*** ^***−1***^ ***)*** ^*4^
*1*	*0.5*	*14.9*	*26.1*	*18.16*	*5.56*	*2.26*	*0.0*	*26.1*	*0.52*	*3.3*	*0.4*	*0.70*	*9.2*	*0.35*	*0.69*
*2*	*0.6*	*19.0*	*21.9*	*15.56*	*5.12*	*2.44*	*0.0*	*28.9*	*0.65*	*3.0*	*0.5*	*0.71*	*8.6*	*0.30*	*0.46*
*3*	*0.6*	*18.8*	*22.8*	*15.85*	*5.29*	*2.47*	*1.0*	*28.3*	*0.59*	*3.0*	*0.5*	*0.70*	*9.6*	*0.34*	*0.58*
*4*	*0.7*	*26.5*	*15.1*	*11.27*	*3.40*	*1.29*	*0.2*	*15.5*	*0.57*	*3.3*	*0.4*	*0.75*	*8.0*	*0.52*	*0.91*
*5*	*0.9*	*28.3*	*13.1*	*9.72*	*3.23*	*1.64*	*0.2*	*15.0*	*0.55*	*3.0*	*0.5*	*0.74*	*9.0*	*0.60*	*1.10*
*6*	*1.0*	*23.1*	*15.2*	*10.36*	*4.21*	*2.86*	*0.0*	*17.0*	*0.58*	*2.5*	*0.7*	*0.68*	*10.2*	*0.60*	*1.03*
*7*	*1.0*	*21.0*	*17.1*	*12.25*	*4.59*	*3.01*	*0.0*	*27.3*	*0.64*	*2.7*	*0.7*	*0.71*	*12.5*	*0.46*	*0.71*
*8*	*1.7*	*31.0*	*11.1*	*7.41*	*2.63*	*1.75*	*0.0*	*17.0*	*0.49*	*2.8*	*0.7*	*0.67*	*12.9*	*0.76*	*1.54*
*9*	*1.9*	*34.9*	*6.8*	*4.47*	*1.90*	*1.42*	*0.0*	*13.2*	*0.26*	*2.3*	*0.7*	*0.66*	*8.5*	*0.64*	*2.49*
*10*	*1.9*	*33.5*	*8.4*	*5.29*	*2.23*	*1.69*	*0.8*	*25.3*	*0.20*	*2.4*	*0.8*	*0.63*	*10.2*	*0.40*	*2.00*
*11*	*2.2*	*30.4*	*11.2*	*7.67*	*2.79*	*1.76*	*0.0*	*25.7*	*0.50*	*2.7*	*0.6*	*0.69*	*17.1*	*0.66*	*1.33*

^*1^Effect of dilution due to NaOH dosing incorporated into the calculation.

^*2^Including scrubbed biofilm and suspended cells.

^*3^Based on total x.

^*4^Based on estimated cellular mass.

**Figure 6:**
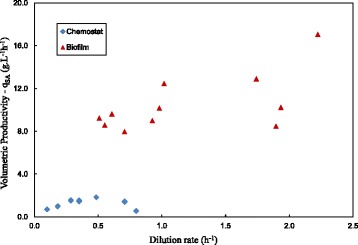
**Chemostat and biofilm volumetric productivities (q**_**SA**_ **) plotted against D.** The comparative q_SA_ values for the chemostat and biofilm reactors indicate the order of magnitude difference in volumetric productivity. Differences can be attributed to total biomass quantities (see Table 1 and Table 2).

To illustrate the significant quantities of biomass present in the biofilm runs, Figure 6 compares the volumetric productivity (q_SA_) of the biofilm runs to that of the of the chemostat results. The order of magnitude difference can be attributed to the total biomass amount in the biofilm reactor varying between 13 and 28 g.L^−1^, while the chemostat runs had a maximum biomass concentration of 2.65 g.L^−1^. For the biofilm runs repeatability of productivity at a given D was not observed. This implies that the amount of biofilm build-up was not repeatable even though steady-state was confirmed. Biofilm coverage of the internal tube area was not complete and large open surfaces were observed. Surfaces without biofilm coverage were continuous without any intermittent biofilm patches.

**Figure 7:**
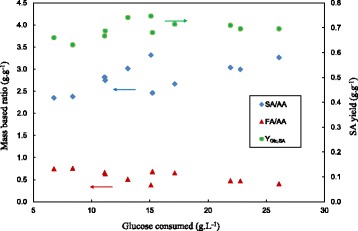
**Biofilm reactor mass-based yield ratios (SA/AA, FA/AA and Y**_**Glc,SA**_ **) plotted against ΔGlc.** When compared to the comparative graph for the chemostat (Figure 3), the Y_Glc,SA_ is significantly higher (up to 50% more). The SA/AA ratio of the biofilm runs is more than double that of the chemostat runs (Figure 3). The FA/AA ratio is close to equimolar (0.77 g.g^−1^) at low ΔGlc but decreases with increasing ΔGlc.

The SA yield and product ratios are given in Figure 7. Since D cannot be used as the independent variable given the variation of biofilm build-up, ΔGlc was chosen to quantify the extent of the fermentation. A striking difference is observed when these results are compared to that of the chemostat runs (Figure 3). The SA/AA ratio of the biofilm runs increased to double that of the chemostat runs. The ratio increased slightly with ΔGlc and reached a maximum value of 3.3 g.g^−1^. This is in direct agreement with the results of Bradfield & Nicol [15] where the ratio was shown to further increase at ΔGlc higher than those achieved in this study. The FA/AA trend is also analogous to the Bradfield & Nicol [15] results where a decrease is observed against ΔGlc. Below a ΔGlc of 10 g.L^−1^ the ratio is close to the equimolar value of 0.77 g.g^−1^ and it is evident that dehydrogenase action (either formate of pyruvate) is negligible at these conditions. The overall SA yield varied between 0.63 g.g^−1^ and 0.74 g.g^−1^ with an increasing trend observed against ΔGlc. This is directly linked to the higher SA/AA ratio and the lower biomass yield.

**Figure 8:**
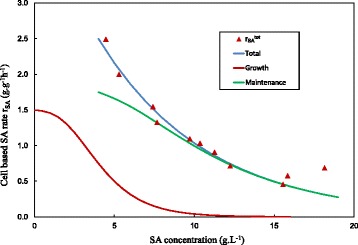
**Breakdown of SA production rate (r**_**SA**_ **).** Measured total rates are given by the red triangles with the empirical fit given by equation (3). The growth contribution to rate is given by the red curve - obtained from the product of the growth function (equation (2)) and Y_x,SA_^true^ (Figure 4). The maintenance contribution to rate is given by the green curve - obtained by subtracting the growth contribution from the total rate.

In Figure 8 the cell based (EPS excluded) SA production rates (r_SA_^tot^) of the biofilm runs are plotted against the SA titre. Results from the EPS separation procedure estimated that the total biomass had a cellular content varying between 20% and 65% (based on a dried fraction – see Table 2). The cellular fraction appeared to be linked to the SA titre in the fermenter where lower SA titres resulted in a lower cellular content. Above a SA titre of 10 g.L^−1^ the cellular fraction only varied between 55% and 65%. The cell based production rate in Figure 8 exhibits a drastic decrease against SA titre. The decrease is also observed when the total biomass based rate is considered (not plotted in Figure 8), but this trend is less severe due to the fact that low SA titre biomass has a lower cellular content.

The curves on Figure 8 attempt to estimate the growth and maintenance contributions to the overall SA production rate (using a cell basis). Since EPS quantification was not performed on the suspended biomass of the chemostat runs, the assumption was made that the biomass consisted predominantly out of cells with negligible EPS present. When the Y_x,SA_^true^ obtained in Figure 4 is multiplied with the growth function of Figure 5 (equation (2)), the growth associated production rate of SA can be obtained as a function of the SA titre (given as the red curve Figure 8). The r_SA_^tot^ in this figure were fitted with the following empirical equation (3):

3 $$ {r}_{SA}^{tot}=4.5 \exp \left(-\frac{C_{SA}}{6.8}\right) $$

The data point at the extreme right of the figure (SA titre =18 g.L^−1^, run 1 in Table 2) was deliberately ignored in the fit since operation at this low D was unstable for extended periods prior to achieving steady-state. The non-growth or maintenance contribution to the SA production rate is calculated by subtracting the growth contribution from the total rate. The resulting maintenance contribution is presented by the green line. The main purpose of the curve fitting is to illustrate that the green line merges with the blue line at relatively low SA titres (10 g.L^−1^ in the figure). This shows that SA production at high SA titres is exclusively maintenance based. The point of convergence of the blue and the green lines is dependent on the growth rate function beyond a SA titre of 10 g.L^−1^. It should be noted that the exact curvature of the growth function tail is an estimated extrapolation. Maharaj *et al.* [13] observed slow biofilm recovery (implying growth) at titres as high as 30 g.L^−1^. This suggests that the tailing asymptote never reaches zero and that extremely slow growth is possible at higher titres, however this contribution to SA production will be negligible.

The biofilm and chemostat data provide clear evidence that maintenance production of SA is the main production mode in the biofilm reactor. The results in Figure 8 also indicate that the maintenance production rate is inhibited at higher acid titres. It is unlikely that the maintenance rate is similar for all cells in the biofilm matrix, since variations in the acid concentrations will exist within the matrix. The acid concentrations will most likely increase with biofilm depth. It is plausible that the deactivation profile presented in Figure 8 is linked to an increasing fraction of metabolically inactive cells, rather than the scenario where all cells are operating at slower metabolic rates when the overall acid titre increases.

Mention needs to be made of the biofilm metabolism versus that of suspended cells. Since the biofilm metabolism is predominantly maintenance based one can rather distinguish between the growth and maintenance metabolism. When the analysis of Bradfield & Nicol [15] is applied, an under-prediction of the SA/AA ratio for the growth metabolism is obtained (associated with NADH ‘losses’), while an over-prediction of the SA/AA ratio is obtained for the maintenance metabolism (associated with NADH ‘gain’). Does this imply that different metabolic pathways are utilized under growth and maintenance conditions? Bradfield & Nicol [15] have shown that additional redox power can be generated by cycling of the pentose phosphate pathway. The NADPH generated in the pentose phosphate pathway cycle needs to be converted to NADH in order to supply redox to the SA pathway. This can be achieved by the transhydrogenase enzyme present in *A. succinogenes*. This presents a plausible explanation for the ‘free’ NADH gained in the maintenance metabolism. Medium contributions were previously considered as the source of for the NADH imbalances, but this can be ruled out given the results of this study where the same medium resulted in opposite redox balance trends.

## Conclusions

Evidence is provided that *A. succinogenes* biofilms have a double advantage when compared to a suspended cell fermenter. The much higher volumetric productivities (q_SA_) of the biofilm runs were expected because of the higher cell densities while the major increase in Y_Glc,SA_ under biofilm conditions was unforeseen. The fact that the same fermenter and same medium resulted in vast differences in the metabolic flux distribution when comparing biofilms to suspended cells, was attributed to metabolic differences between the maintenance and growth modes. It was shown that sessile cells operate predominantly under maintenance conditions and that the metabolic activity of the sessile cell populations (EPS excluded) decreased at higher acid titres.

The quantitative rate data provided in this study will be very helpful for future fermenter designs where different biofilm support geometries will be employed. The specific growth function (Equation (2)) derived from the chemostat runs are in close agreement to the results obtained from other batch studies. The severity of growth inhibition at relatively low acid titres should be considered when accumulating biofilm cells. Similar to Bradfield & Nicol [15], the results of this study suggest that higher SA yields are associated with higher SA titres. These advantages will be counteracted by the maintenance inhibition at higher SA titres where productivity will be lower. It might be possible to side-step this trade-off by utilising non-steady state production of SA. It is suspected that the metabolic response of *A. succinogenes* to increasing acid titres is fairly slow since the non-steady response obtained when lowering D always result in an overshoot of the SA titre. In this regard repeat batches on the same biofilm might be able to achieve faster maintenance rates by reducing prolonged exposure to high acid titres. This remains to be tested.

From a general biofilm reactor perspective this study has shed new light on the possible advantages of single culture biofilm fermentation. The observed variations in the flux distributions between the sessile and planktonic cells of this organism might apply to other biofilm producers. High cell densities are naturally achieved by biofilm-forming species and cell retainment equipment is accordingly redundant. This well-established advantage in combination with a higher carbon yields will contribute to the favour of the biofilm fermenter in years to come.

## Methods

### Microorganism and growth medium

*A. succinogenes* 130Z (DSM 22257 or ATCC 55618) was acquired from the German Collection of Microorganisms and Cell Cultures (DSMZ). Seed cultures in 30-mL McCartney bottles containing 15 mL sterilised TSB were incubated for 16–24 hours at 37°C and 150 rpm prior to use (for stock cultures or reactor inoculation). Short term (<3 weeks) stock cultures were stored at 4°C in tryptone soy broth (TSB) from Merck KgaA (Darmstadt, Germany). For long term storage (>3 weeks), 1 g of 66.6% glycerol solutions were inoculated with 0.5 mL stock culture and stored at -40°C.

### Medium

All chemicals were sourced from Merck KgaA (Darmstadt, Germany), unless specified differently. The growth medium and phosphate buffer used for the experimental runs were the same as that used by Bradfield & Nicol [15]. The growth medium consisted of (g.L^−1^): YE: 6; clarified CSL [15] (Sigma–Aldrich, St. Louis, Missouri): 10; NaCl: 1.0, MgCl_2_6H_2_O: 0.2, CaCl_2_2H_2_O: 0.2, sodium acetate: 1.36, Na_2_S9H_2_O: 0.16 (for anaerobic conditions) and 1 mL.L^−1^ Antifoam A (Sigma-Aldrich. St. Louis, Missouri). The phosphate buffer consisted of (g.L^−1^): KH_2_PO_4_: 3.2 and K_2_HPO_4_: 1.6. A D-glucose (Futaste Pharmaceutical Co. Ltd., Shandong, China) concentration of 40 g.L^−1^ was used for all fermentations.

The growth medium was diluted in 8 L of distilled water (10-L bottle), the phosphate buffer in 0.5 L of distilled water (1-L bottle) and Glc in 1.5 L of distilled water (2-L bottle) and separately sterilised by autoclaving at 121°C for 40 min. Prior to use, the solutions were left to cool to room temperature to prevent unwanted reactions amongst the components, after which the Glc solution and phosphate buffer were aseptically added to the growth medium.

### Bioreactor

**Figure 9:**
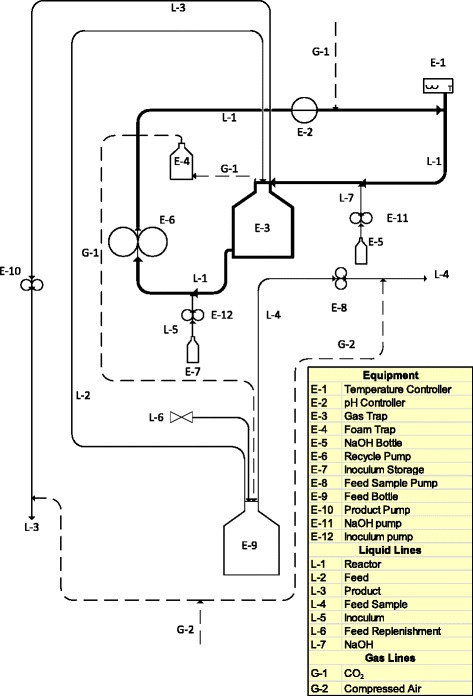
**The bioreactor setup used for both the chemostat and biofilm experiments.** The reactor section is shown in bold with an in-line gas trap. The reactor section consists of a 3 mm silicone tube (approximately 5 m length) with an active volume of 50–60 mL, depending on the liquid level in the gas trap.

The experimental setup used in the investigation is shown in Figure 9; the reactor section is shown in bold and contains an in-line gas trap to continuously remove CO_2_ from the reactor preventing CO_2_ accumulation. The reactor consisted of a 3 mm silicone tube of approximately 5 m length with an active volume of 50 mL – 60 mL, depending on the amount of gas holdup in the reactor. A feed line, an NaOH dosing line, an inoculation line, a CO_2_ (Afrox, Johannesburg, South Africa) line and a product line were connected to the reactor section as shown in Figure 9. Compressed air was connected to the feed sample and the product lines, directly after the peristaltic pumps, to establish positive pressure which assisted in maintaining aseptic conditions. Anaerobic conditions were maintained by the peristaltic pumps which prevented inflow of air from the compressed air lines.

Temperature was controlled at 37°C using a hotplate coupled to a thermocouple, housed in an aluminium sheath connected within the reactor. pH was controlled at 6.80 by dosing unsterilized 10 M NaOH through a peristaltic pump 120U (Watson Marlow, Falmouth, UK) with a relay connected to a Liquiline CM442 controller (Endress + Hauser, Gerlingen, Germany). The controller was connected to a Tophit CPS471D ISFET pH probe (Endress + Hauser, Gerlingen, Germany) housed within an in-line stainless-steel holder. The CO_2_ flowrate to the reactor was continuously controlled at a constant flowrate of 6 mL.min^−1^ (±0.1 vvm) with a Brooks SLA5850S thermal mass flow controller (Brooks Instrument, Hatfield, Pennsylvania). The CM442 and the SLA5850S were connected to a data logging system NI USB-6008 (National Instruments, Austin, Texas) whereby pH, relay position, temperature and CO_2_ flowrate were recorded continuously during fermentations.

The shear velocities (the quotient of the volumetric recycle rate (m^3^.s^−1^) and the cross sectional area (m^2^) of the tube) used for the experiments were 1.83 m.s^−1^ (Re ≈ 7800, i.e. fully turbulent flow) for the chemostat experiments and 0.09 m.s^−1^ (Re ≈ 400, i.e. fully laminar flow) for the biofilm experiments. Due to the high recycle rate, as compared with the reactor through flow, it was assumed that the reactor section acted as a perfectly mixed reactor with negligible axial and radial concentration profiles, because typical ratios of recycle flow to the reactor through flow were between 90 and 8850 [33]. This was confirmed by residence time distribution tests performed *in situ* by applying a pulse change to the NaOH flowrate for pH control and measuring the pH change over time.

The time averaged rate of NaOH dosed, for pH control, was monitored continuously and used as an indication of steady-state in the system. To ensure steady-state in the reactor, the effluent was analysed twice with at least two volume turnovers between samples, when the rate of NaOH dosing fluctuated less than 5%.

### Product analysis

The bacterium produces four distinct metabolic products: SA, AA, FA and ethanol [21]. The concentrations of which were measured, along with residual Glc, using an Infinity 1260 high-performance liquid chromatograph (Agilent Technologies, Santa Clara, California) with an Aminex HPX-87H ion-exclusion organic acid column (Bio-Rad Laboratories, Berkeley, California). The column was pre-calibrated using > 99% purity standards sourced from Merck KgaA (Darmstadt, Germany) and Sigma–Aldrich (St. Louis, Missouri).

### Biomass quantification

The relationship between biomass and absorbance was determined by measuring the absorbance (ABS_660_), at a wavelength of 660 nm (CE 1021 Spectrophotometer, Cecil Instruments, Cambridge, UK), of 46 individual samples from various reaction conditions and biomass concentrations. The samples included suspended and total biomass measurements (Measurement of total biomass section). The samples were washed twice with distilled water, re-suspended in distilled water, the ABS_660_ was measured and the samples were dried overnight at 70°C. It was determined that an absorbance value of 1 relates to 645 mg L^−1^ of biomass with a correlation coefficient of 0.97. The biomass concentrations were inferred from this relationship and ABS_660_ measurements.

### Measurement of total biomass

Biofilm quantification was achieved by physically removing the biofilm from the internal surface of the bioreactor. All feed to the bioreactor as well as recycling in the bioreactor was ceased and using mechanical friction through the soft silicone tubing, the biofilm was loosened and subsequently removed by increased shear (1.83 m.s^−1^). To ensure total removal of the biofilm, the reactor volume was emptied into the gas trap to prepare the silicone tubing for inspection. The process was repeated until all observable traces of biofilm were removed from the tubing. During this process, the removed biofilm was thoroughly mixed with the medium due to the significant shear and turbulence in the reactor.

In order to keep the reactor ready for subsequent experiments, without the need for a complete restart, the, reactor was incompletely drained after removal of the biofilm. The silicone-tube section of the reactor was emptied into the gas-trap section of the reactor. A sample of approximately 40 mL was taken from the gas trap and the biomass concentration was determined by ABS_660_ (Biomass quantification section). The removed volume was then replaced with clean feed to allow for the re-initialisation of the reactor for the next run.

### EPS quantification

EPS quantification was done by alkaline hydrolysis and subsequent removal of the EPS fraction from the biofilm by dissolution in distilled water [34]. For preliminary tests, the samples were treated with pH 9 and pH 11 buffers, using the procedure described below. However it was found that the difference in results between the two pH buffers were negligible, and therefore only the pH 9 buffer was subsequently used. The pH 9 buffer consisted of 1 g.L^−1^ KH_2_PO_4_ and 79 g.L^−1^ K_2_HPO_4_, and the pH 11 buffer of 0.01 g.L_−_^−1^ KH_2_PO_4_ and 79 g.L^−1^ K_2_HPO_4_ (ratios of 0.0162 mol KH_2_PO_4_.(mol K_2_HPO_4_)^−1^ and 0.000162 mol KH_2_PO_4_.(mol K_2_HPO_4_)^−1^, respectively). The buffers were prepared by initially adding the KH_2_PO_4_ to a 1-L mixing vial and then adding the K_2_HPO_4_ while monitoring the pH (Alpha pH 190 pH controller, Eutech Instruments, Singapore).

For EPS removal, a known volume of the total biomass sample after ABS_660_ measurement (Measurement of total biomass section) was centrifuged and re-suspended in a known volume of the required buffer solution (pH 9 or pH 11). The sample was ultrasonicated for 30 min (UMC 2, Integral Systems, Johannesburg, South Africa), centrifuged and washed twice with distilled water. Finally, the washed sample was resuspended in a known volume of distilled water and the ABS_660_ was measured. The ABS_660_ values of the pre-treated and treated samples were converted to biomass concentrations and adjusted for dilution. The ratio of concentrations before and after treatment was assumed to represent the fraction of cellular-to-total biomass (including EPS).

## Abbreviations

SA: Succinic acid
EPS: Extracellular polymeric substance
μ: Specific growth rate (h^−1^)
Glc: Glucose
Y_Glc,SA_: Yield of SA on Glc (g.g^−1^)
ΔGlc: Glucose consumed (g.L^−1^)
AA: Acetic acid
FA: Formic acid
x: Biomass
Y_x,SA_^true^: Growth-associated yield coefficient of SA on biomass (g.g^−1^)
D: Dilution rate (h^−1^)
μ_max_: Maximum specific growth rate (h^−1^)
q_SA_: Volumetric production rate of SA (g.L^−1^.h^−1^)
r_SA_: Specific production rate of SA (g.g^−1^ h^−1^)
SA/AA: SA to AA ratio (g.g^−1^)
FA/AA: FA to AA ratio (g.g^−1^)
m_SA_: Maintenance production of SA (g.g^−1^.h^−1^)
r_SA_^tot^: Total specific SA production rate (g.g^−1^.h^−1^)
C_SA_: SA titre (g.L^−1^)
YE: Yeast extract
CSL: Corn steep liquor
NADH: Nicotinamide adenine dinucleotide
NADPH: Nicotinamide adenine dinucleotide phosphate
ABS_660_: Absorbance of biomass suspension at a wavelength of 660 nm (−)
